# Hyperprolactinemia in patients taking antipsychotics: the importance of a shared approach between psychiatry and endocrinology

**DOI:** 10.1192/j.eurpsy.2023.1138

**Published:** 2023-07-19

**Authors:** M. Mousinho, A. Gouveia, B. Pimentel

**Affiliations:** 1 Psychiatry, Unidade Local de Saúde do Baixo Alentejo, Beja; 2 Centro Hospitalar Lisboa Central, Lisboa, Portugal

## Abstract

**Introduction:**

Hyperprolactinemia is a commonly encountered adverse effect of antipsychotic medication. Short and long-term repercussions of high prolactin, such as amenorrhea, sexual dysfunction, osteopenia and increased cardiovascular risk carry significant burden and may compromise therapeutic adherence. Despite its serious practical implications, hyperprolactinemia is still underscreened and its management neglected.

**Objectives:**

To review current clinical guidelines regarding the management of hyperprolactinemia associated with the use of antipsychotics, reflecting upon the importance and need to share the management of this risk with an endocrinology expert.

**Methods:**

We performed a literature review to identify clinical guidelines containing specific recommendations for antipsychotic-induced hyperprolactinemia (British Association of Psychopharmacology [BAP], NICE, Maudsley Prescribing Guidelines, Royal Australian and New Zealand College of Psychiatrists), published over the last ten years, with a particular focus on its physical risks.

**Results:**

Most guidelines do not recommend routine monitoring of prolactin levels in asymptomatic patients. NICE and BAP guidelines have suggested measuring the baseline prolactin level, but have not specified follow-up monitoring, while Maudsley guidelines have. Management strategies depend on factors such as sex, age, as well as the clinical manifestations that ensue. Different treatment strategies have been described, such as decreasing the dose of the antipsychotic, switching antipsychotics, adding aripiprazole or adding dopaminergic agonists. Referral to an endocrinology specialist should be made if the aetiology is unclear, prolactin levels continue to rise despite some intervention, the hyperprolactinaemia is severe (>3000 mIU/L) or there is suspected/confirmed pituitary adenoma. Further physical implications of having hyperprolactin are to be dressed by the endocrinology expert, namely those on bone metabolism, gonodal function and cancer risk.

**Conclusions:**

Given the widespread use of antipsychotics and the need to have psychotic patients stabilized (sometimes with a lack of effective alternative), early detection and shared management of hyperprolactinemia are instrumental towards assisting both clinician’s and patients’ decision-making, be it towards lowering prolactin levels or managing its risk without compromising the antipsychotic’s efficacy.

**Disclosure of Interest:**

None Declared

